# Using behavioral theory and shared decision-making to understand clinical trial recruitment: interviews with trial recruiters

**DOI:** 10.1186/s13063-021-05257-x

**Published:** 2021-04-21

**Authors:** Jamie C. Brehaut, Carolina Lavin Venegas, Natasha Hudek, Justin Presseau, Kelly Carroll, Marc Rodger

**Affiliations:** 1 Clinical Epidemiology Program, Ottawa Hospital Research Institute, 501 Smyth Road, Box 201B, Ottawa, ON K1H 8L6 Canada; 2 School of Epidemiology and Public Health, University of Ottawa, Ottawa, Ontario Canada; 3 The Ottawa Hospital, Ottawa, Ontario Canada

**Keywords:** Theoretical Domains Framework, Shared decision-making, Recruitment

## Abstract

**Background:**

Clinical trial recruitment is a continuing challenge for medical researchers. Previous efforts to improve study recruitment have rarely been informed by theories of human decision making and behavior change. We investigate the trial recruitment strategies reported by study recruiters, guided by two influential theoretical frameworks: shared decision-making (SDM) and the Theoretical Domains Framework (TDF) in order to explore the utility of these frameworks in trial recruitment.

**Methods:**

We interviewed all nine active study recruiters from a multi-site, open-label pilot trial assessing the feasibility of a large-scale randomized trial. Recruiters were primarily nurses or master's-level research assistants with a range of 3 to 30 years of experience. The semi-structured interviews included questions about the typical recruitment encounter, questions concerning the main components of SDM (e.g. verifying understanding, directive vs. non-directive style), and questions investigating the barriers to and drivers of their recruitment activities, based on the TDF. We used directed content analysis to code quotations into TDF domains, followed by inductive thematic analysis to code quotations into sub-themes within domains and overarching themes across TDF domains. Responses to questions related to SDM were aggregated according to level of endorsement and informed the thematic analysis.

**Results:**

The analysis helped to identify 28 sub-themes across 11 domains. The sub-themes were organized into six overarching themes: coordinating between people, providing guidance to recruiters about challenges, providing resources to recruiters, optimizing study flow, guiding the recruitment decision, and emphasizing the benefits to participation. The SDM analysis revealed recruiters were able to view recruitment interactions as successful even when enrollment did not proceed, and most recruiters took a non-directive (i.e. providing patients with balanced information on available options) or mixed approach over a directive approach (i.e. focus on enrolling patient in study). Most of the core SDM constructs were frequently endorsed.

**Conclusions:**

Identified sub-themes can be linked to TDF domains for which effective behavior change interventions are known, yielding interventions that can be evaluated as to whether they improve recruitment. Despite having no formal training in shared decision-making, study recruiters reported practices consistent with many elements of SDM. The development of SDM training materials specific to trial recruitment could improve the informed decision-making process for patients.

**Supplementary Information:**

The online version contains supplementary material available at 10.1186/s13063-021-05257-x.

## Background

Recruiting and retaining research participants remains a central challenge for clinical research. In the USA, 40% of the National Cancer Institute-funded trials are discontinued, nearly half because of recruitment issues [1]. In Canada, the Canadian Cancer Clinical Trials Network data shows extremely low trial participation rates among patients treated at regional cancer centers (8%) and community hospitals (1.6%) [2]. These challenges are not limited to cancer trials. One exploration of diverse discontinued trials found that nearly 40% cited poor recruitment as the primary cause [3]. Another study found that 18% of trials were terminated because of poor recruitment, corresponding to nearly 50,000 patients participating in trials that could not adequately answer their primary research question [4]. One analysis calculated up to 10,000 unnecessary deaths in the USA attributable to delays in recruitment to a practice changing randomized controlled trial in patients with acute myocardial infarction [5, 6]. Low recruitment and retention increases the costs of research, leads to research waste, lowers the validity of studies, slows innovation, and prevents timely implementation of effective care.

Systematic reviews have noted poor or highly variable quality of studies comparing strategies for improving recruitment and retention in clinical trials [5, 7–11], while only providing limited guidance for trialists about individual study elements (e.g. telephone reminders, open trial designs) that may improve recruitment [9]. Extensive efforts to build the evidence base around such study elements are ongoing, including initiatives to coordinate and develop capacity for nesting recruitment interventions within other trials [9], and the development of systematic qualitative approaches to identifying site-specific barriers and drivers of recruitment [5, 12–14]. Such efforts have not typically been guided by decision-making and behavior change theories, theories shown to be useful for designing health behavior and implementation interventions.

Improved recruitment could come from considering the recruitment process through a lens of two influential theoretical frameworks. First, participation in clinical research is often a difficult decision that could benefit from *shared decision-making (SDM)*, a framework originally designed to help people work systematically through difficult treatment decisions, often with decision-making tools called patient decision aids that seek to achieve the clear goals of improving knowledge of key aspects of the decision, understanding outcome probabilities, and ensuring a match between outcome preferences and the choice made [15–17]. More recently, SDM principles are being applied to choices related to clinical trial involvement [18–22], such as consent [18–21] and participation decisions [22–24]. This framework provides a fundamentally different model about what informed consent and recruitment could look like, emphasizing shared, deliberative decision-making between patient and provider, and improved quality of decision-maker experience by ensuring understanding of the probability of relevant outcomes and a match between outcomes most valued by the decision maker and the decision made. Second, we propose that recruitment involves different behaviors by multiple individuals. In our study, we focus on recruiter behaviors during recruitment interviews. The *Theoretical Domains Framework (TDF)* [25–32] organizes over 100 constructs associated with behavior into 12 domains that can be explored through interviews with stakeholders. It has been used successfully in a wide range of clinical areas and is the most comprehensive means of eliciting barriers to and drivers of health behaviors [33]. Furthermore, by identifying which domains are relevant to recruitment, we can design interventions specifically tailored to these challenges based on the now considerable literature on what behavior change techniques are effective for intervening on these domains [33, 34].

As a first step towards developing a theory-guided approach to recruitment strategy development, this study seeks to explore the utility of SDM and the TDF for understanding and organizing the existing strategies reported used by recruiters during recruitment interactions. We interviewed recruiters for one multi-site trial, with the objectives of (1) identifying the elements of SDM recruiters currently use and (2) identifying the full range of barriers and drivers relevant to study recruitment in the target trial according to the TDF.

## Methods

### Design

This was a theory-informed, semi-structured interview study of non-physician recruiters for a single, multi-site clinical trial. Study elements are reported according to COnsolidated criteria for REporting Qualitative research (COREQ) standards [35] (Additional file 1).

### Target trial

The **S**t**A**tins for **V**enous **E**vent **R**eduction (SAVER) study is a randomized, open-label pilot study assessing feasibility of a large-scale trial designed to evaluate if generic rosuvastatin reduces the risk of recurrent venous thromboembolism in patients with symptomatic major venous thromboembolism (NCT02679664). This study is taking place in five Canadian centers part of the Canadian Venous Thromboembolism Clinical Trials and Outcomes Research Network (www.canvector.ca). Eligible consenting patients who develop acute, symptomatic, and objectively confirmed venous thromboembolism (proximal leg vein deep vein thrombosis and/or pulmonary embolism) are randomized to a treatment group (receiving rosuvastatin 20 mg daily in addition to usual care anticoagulation) or standard of care control group (standard anticoagulant treatment). After potential eligibility is determined by the study team, patients are approached by their physician and, if interested, referred to study coordinators (recruiters) for further information and consenting. The pilot trial consists of up to five study contacts over 6 months: screening, randomization, telephone follow-ups (90 days, drug group only), and final study visit (180 days). The primary outcome for the pilot trial is feasibility of recruitment for the full trial.

### Participants

Our sample frame included recruiters from all five Canadian sites of the SAVER pilot trial (*n* = 10 recruiters). As part of a network involved in many such trials, these recruiters (many nurses or master's-level research assistants) had considerable experience recruiting and spent a substantial portion of their day engaged in recruitment activities. The SAVER investigator team was interested in identifying barriers to address and strategies to use prior to rollout for the larger study, hence the opportunity to have discussions with recruiters about their recruitment strategies.

### Interview guide

The interview (see Additional file 2) included four sections: (1) ice-breakers asking interviewees how long and for what trials they have recruited; (2) asking interviewees to describe a typical patient recruitment encounter (e.g. how they approach the patient, materials they use, factors they consider important to recruitment) [18, 19]; (3) questions regarding constructs central to SDM, such as verifying understanding, clarifying outcome preferences, and taking a directive (i.e. focused on participation) vs. non-directive (i.e. focused on helping with their decision) approach; and (4) questions focused on understanding barriers and drivers to pilot trial recruitment, based on the TDF [25]. For the TDF portion of the interview guide, and informed by the AACTT principle for specifying target behaviors [36], we defined the behavior under investigation as recruitment (Action) of patients eligible for the SAVER trial (Target), by the recruiters (Actors), in-hospital (Context), and during the 30-day window of eligibility for the SAVER pilot (Time). The interview was designed to be completed in approximately 1 h.

### Procedure

The Ottawa Health Science Network Research Ethics Board provided ethics approval (20170690-01H). The interview guide was pilot-tested with three local, non-SAVER recruiters to examine the appropriateness, flow, and robustness of the guide, modifying after each session. A purposive sample consisting of all active recruiters for the SAVER pilot were sent an email from a SAVER principal investigator (MR) notifying them of the forthcoming interview study, followed by a formal invitation email from the interview study principal investigator (JCB) which included a participant information document and description of the study. A maximum of three follow-up emails were sent to non-responders at 1-week intervals, asking them to contact the study coordinator to participate. Interviews were conducted one-on-one, in person (written consent) or over the phone (verbal consent) in a private room at an academic hospital with a female master's-level research coordinator (KC) experienced in conducting qualitative research. Interviews were digitally, audio-recorded, transcribed, and anonymized. Interviewer notes were also taken during each interview. Participants were offered $40 as a token of appreciation.

### Analysis

First, we employed a directed content analysis to assign quotations to TDF domains. We then conducted two separate inductive thematic analyses to identify sub-themes within TDF domains and again to identify overarching themes across TDF domains. Analysis was carried out after all interviews were completed. Sub-themes identified from previous interviews were continually revisited to identify new sub-themes and to allow for the revision, combination, or separation of sub-themes [37, 38]. Two raters (CLV, KC) independently reviewed and coded the first two interviews and then met to achieve consensus on initial coding and code categories. Once a basic coding scheme was agreed upon, raters continued with analysis, meeting regularly for consensus and to discuss new sub-themes. Sub-themes were then grouped according to categories for action (e.g. coordinating between people, providing guidance to recruiters). While we were limited to the number of recruiters available to us, we planned to assess theme and sub-theme saturation at the end of the interview process using the same principle as the 10 plus 3 rule typical of TDF research [39]. We examined the occurrence of any new themes or sub-themes in the final 3 interviews.

SDM-related questions targeted 10 concepts from the SDM literature (e.g. assessing patients’ decision-making needs, verifying understanding) to explore the extent these concepts were part of these recruiters’ process. While we framed many of the questions as simple endorsements (“how often do your interactions with patients involve the following activities”), these items generated discussion about process as well; therefore, while we report overall rate of endorsement of each item, these discussions also informed our thematic analysis. All data coding and analysis was conducted using Microsoft Excel (version 2010).

## Results

We conducted interviews with all nine active recruiters from the five SAVER sites (one had not recruited for this study). The sample included both males (*n* = 3) and females (*n* = 6) who had between 3 and 30 years of experience in recruiting for trials. Interview duration averaged approximately 1 h (mean 63 min; range 42–79 min).

### TDF themes

Analysis of barriers and drivers involved quotations being assigned to 11 of the 12 TDF domains. Subsequent inductive analysis led to the identification of 28 sub-themes across six overarching themes: (1) coordinating between people, (2) providing guidance to recruiters, (3) providing resources to recruiters, (4) optimizing study flow, (5) guiding the recruitment discussion, and (6) emphasizing the benefits to participation (Table 1).

1. “Coordinating between people”—All recruiters touched on the notion that good coordination between recruiters and physicians was important, as was having the physicians actively involved in the recruitment process. All recruiters discussed the need for coordination with others (i.e. other recruiters, clinical team members, screeners) who can identify eligible patients before the physician or recruiter speaks with them. These sub-themes primarily fell into the Behavioral Regulation or Social Influences domains, domains receptive to behavior change interventions such as problem solving (i.e. stakeholders developing strategies to overcome barriers and/or increase drivers) and social support (i.e. eliciting practical help from colleagues) [40]. One barrier identified within this theme was a single mention of physicians having their own idiosyncratic inclusion criteria (i.e. not recommending patients over a certain age when not a formal exclusion criterion) and that such physician strategies took recruitment out of recruiter’s hands (Beliefs about Capabilities domain). Another barrier mentioned by one recruiter involved recruiting for multiple competing studies, leading to a conflict (Motivation and Goals). While these barriers were only identified by single individuals, both relevant domains may be successfully targeted by things like incentives for physicians or recruiters.
2. “Providing guidance to recruiters about challenges”—Almost all recruiters indicated they did not receive formal training, and several felt they would have benefitted from such training. Some reported feeling discouraged when recruitment was not successful, or deciding not to recruit for various patient-related reasons (i.e. patient not understanding or behaving aggressively). These sub-themes were primarily coded into the Skills and Memory, Attention, & Decision Process domains. Skills can be targeted by behavior change interventions like providing instruction and behavioral practice/rehearsal, while Memory, Attention, & Decision Process can be targeted by using prompts or cues intended to elicit the desired behavior) [40].
3. “Providing resources to recruiters”—Nearly all recruiters indicated being motivated by having recruitment goals and knowing their recruitment numbers, while some also indicated that knowing the numbers of other recruiters or sites brought out a motivating competitiveness. Many recruiters felt having a backup recruiter, access to appropriate space for the consent conversation, and appropriate reminders to follow-up with potential participants were also essential resources. The majority of these sub-themes fit into the Motivation & Goals, Knowledge, and Environmental Context/Resources domains. These domains are receptive to behavior change interventions such as goal setting and feedback, information about social and environmental consequences of performing the behavior, and restructuring the physical environment [40]. Some recruiters made use of visual aids already present in the recruitment space (i.e. posters on thrombosis), while others noted lack of ethics approval as a barrier to using study-developed visual aids (Behavioral Regulation domain). The use of visual aids could be facilitated by ensuring ethics approved materials are available at all sites.
4. “Optimizing study flow”—This theme includes several barriers within the Nature of the Behavior domain. Some recruiters felt that allowing the consent form to go home with potential participants was a barrier, while others required a consent decision right away and equally felt that was a barrier. The lack of consensus on this issue suggests an area where more research is needed. Other barriers identified by some recruiters were time pressure (1-month window) and the tests required to assess eligibility (which interfere with this timeframe), suggesting a clear plan to identify the best opportunities to contact patients may improve recruitment.
5. “Guiding the recruitment decision”—The drivers from this theme fell under the Behavioral Regulation domain. All recruiters were aware of the importance of patient understanding of study information; they asked and encouraged questions from patients to ensure comprehension. Several recruiters also indicated that not rushing a decision from patients and emphasizing the minimal burden of participation facilitated successful recruitment. Behavior regulation strategies may help recruitment efforts (e.g. self-monitoring), but may also require consideration of the ethical implications of framing information in different ways, ideas that stem from the SDM model.
6. “Emphasizing the benefits of participation”—The final theme included several drivers related to Behavioral Regulation that could be incorporated into the consent discussion. Most recruiters felt highlighting certain aspects of the study facilitated recruitment; such as patients having a nurse available during the study and the study design having no placebo group. More general benefits that recruiters highlighted to encourage participation included patients having an increased sense of control over their condition and that they would be helping others.

**Table 1:** Themes and sub-themes identified in interviews of recruiters to the SAVER study. Sub-themes categorized according to Barriers (B) and Drivers (D), including frequency and relevant TDF domain (*n* = 9)

Theme	Sub-theme	Frequency (out of 9)	TDF Domain	Representative Quote
Coordinating between people	Coordination between other recruiters and physicians is essential to success	D: 9	Behavioral regulation	“There’s a group of us and we are on a rotation schedule. So the day before you screen through the clinics to identify potential patients and that one person does it so that it’s not 15 coordinators going through the same list of patients.”
	Physicians are seen as essential to recruitment; especially reminding physicians about the study and physician buy-in	D: 9	Behavioral regulation; social influences	“I find I’m more successful at recruiting patients into research with certain physicians.” “So the physicians who are first asking the patients if they would like to talk to us. If they have bought into the study then I see a higher percentage of patients who will ultimately say yes…”
	Recruiters rely on others to initially approach patients	D: 9	Social influences	“Having people that are educated enough on the inclusion/exclusion to identify these people is key because we cannot speak to somebody if they have not identified them in the first place.”
	Having a (non-physician) screener identify patients to recruiters	D: 7	Social influences	“We actually have another casual research nurse. So yeah they are screening clinics the day before and highlighting any possible potential patients for the, the person working the following day.”
	Physicians sometimes have their own inclusion/exclusion criteria (e.g. age)	B: 1	Beliefs about capabilities	“They [physicians] have like arbitrary kind of cut-offs for patients as far as maybe age like I’m not gonna [sic] refer anyone over 70 years old or, you know, something like that which is not an exclusion.”
	Competing studies can result in non-recruitment	B: 1	Motivation & goals	“When we have the big pharma studies they it’s more of a, a high I guess to put patients into those studies in comparison to a smaller study like SAVER.”
Providing guidance to recruiters about challenges	Recruiters say they did not receive formal training	B: 7	Skills	“I was trained on the protocol and trained on like so that I guess would be given the information, the skeleton information to do the consent process. But not really trained.”
	Recruiters report feeling frustration/disappointment when recruitment does not go well	B: 6	Emotion; beliefs about consequences	“You really get down on yourself. You feel like a failure, a bit of a fraud in kind of what you are doing.”
	Recruiters feel they would benefit from training	B: 5	Skills	“I think it’s a good idea for people that are newer to go in, of course, with, with somebody that’s done it before to, to just to listen to the spiel.”
	Deciding not to recruit because they feel patient is not understanding the study	B: 2	Memory, attention, decision process	“If you start to get a feeling based on the questions that they are asking you or the responses they are giving that maybe they cannot really be quality consenting.”
	Deciding not to recruit because patient is unpleasant/aggressive	B: 2	Memory, attention, decision process	“… they are violent in the ER. You know what I mean? … not to say that we should not recruit those patients but it can be difficult from a coordinator standpoint to include them in, in the study.”
Providing resources to recruiters	Having recruitment goals is motivating	D: 8	Motivation & goals	“So we have our monthly targets.”
	Knowing personal recruitment numbers is helpful	D: 8	Knowledge	“There’s tons of information about the number of patients you are screening/month, the number of patients you, you are recruiting/month which is all fairly accessible.”
	Having a backup recruiter is important	D: 7	Environmental context/resources	“…and we have backups like every study, every person has backup so like I’ll back up her study, she’ll back up my studies…”
	Recruiters feel they have appropriate reminders in place, and that such reminders are essential	D: 5	Memory, attention, decision process	“Once they leave the, once they leave the hospital then it’s all up to the coordinator to remember to follow up with them. So I, I try to use Outlook to be honest with you. I put, I’ll put reminders.”
	Having access to appropriate room/space for recruitment is important	D: 7	Environmental context/resources	“We have a really good clinic upstairs so everyone has this, this availability. So we actually have 2 rooms that are set up for research, 2 clinical rooms…”
	Competing with other recruiters/sites is motivating	D: 3	Social influences	“If I see that external sites are doing better than yeah I have that competitive kind of side of me that wants to increase numbers.”
	Insufficient time for recruitment	B: 3	Environmental context/resources	“I think the only resource that I’m lacking is time.”
	Inadequate access to space	B: 2	Environmental context/resources	“We do not have extra space in our clinic like another room that we can take patients to, to go through consent and stuff like that.”
	Using visual aids (i.e. posters) to facilitate patient understanding	D: 2	Behavioral regulation	“So we have a visual diagram that … describes post thrombotic syndrome and has pictures. So that is a tool that I often point to for the patient. I always find that visual aids are helpful and one of the secondary outcomes that they are looking for in this study is preventing that.”
	Visual aids were developed but unused as not REB approved	B: 2	Behavioral regulation	“So he created a, which he will have told you, is a 2-page study aid. I do not bring it in because we did not get REB approval for using it, but I basically highlight it in my conversation…”
	Physical location noted as a barrier until changed for co-location with study physicians	B/D: 1	Environmental context/resources	“Now we are all in one location and it seems to be a lot better… it’s much easier to speak about potential patients if you meet them in the hallway where it wasn’t necessarily happening before.”
Optimizing study flow	Allowing the consent forms to go home for a later decision	B:3;D:1	Nature of the behavior	“So those patients sometimes will take it home and come back which I, I do not like because I think there is—the ability to recruit those patients becomes a lot harder once they have, they have left and they have to come back.”
	Time pressure felt by recruiters (1-month window)	B: 3	Nature of the behavior	“Well you have to consent patients within one month of diagnosis of clot.”
	Requiring a consent decision right away	B: 2	Nature of the behavior	“The problem with SAVER and some of these studies is that you have to consent now. You do not, you know, we cannot push it back a week.”
	Tests required to assess eligibility sometimes interfere with time window	B: 1	Nature of the behavior	“SAVER trial in particular there’s a few hoops you have to jump through. You have to have some baseline blood work to see that they are eligible.”
Guiding the recruitment discussion	Recruiters are aware of the importance of patients’ understanding and ask questions to ensure they understand (informed consent)	D: 9	Behavioral regulation	“I think the most one of the most important things for retention in recruitment would be patients’ understanding … to determine whether they actually understand what they are reading is obviously very important.”
	Emphasizing the minimal burden for patients is a successful recruitment strategy	D: 4	Behavioral regulation	“I also try and stress on my end the way that I’m flexible to make it not a burden on them. So if it, you know, follow-ups are all done by telephone and you’ll not have to come back for extra visits that tends to be the biggest burden for patients is having to return to the hospital …”
	Recruiters feel that not rushing a decision from patients is important	D: 4	Behavioral regulation	**“**I would try to avoid rushing the patient. I, you never want to make them feel like they are rushed at all…”
Emphasizing the benefits of participation	Informing patients that a nurse will be readily available during the study facilitates consent	D: 7	Behavioral regulation	“…that they have someone to call if they have any concerns and we can get them into clinic more much more quickly.”
	Recruiters suggest participation will provide patient with a sense of control over their condition	D: 5	Behavioral regulation	“And sometimes when you cannot control your disease or your illness this is a way of controlling. You get to choose. I’ve sold it that way too.”
	Reminding patients that participation would help others	D: 4	Behavioral regulation	“That they are taking part in something where they could truly benefit and it’s also helpful for people down the road who will develop VTE.”
	Study design facilitates recruitment (i.e. no placebo)	D: 2	Beliefs about capabilities	“Something that made me more confident is the fact that we are not using placebo in this study.”

### SDM themes

We asked recruiters a variety of questions derived from the SDM perspective; first, whether a recruitment interaction could be successful if it did not end in recruitment. From a trialist basis, recruitment is clearly the goal, and a choice not to participate might be seen as a failure. From a SDM perspective however, the goal of the conversation is to help people make the decision that is right for them, whether it ends in recruitment or not. All recruiters indicated that a recruitment interaction could be successful without recruitment. However, their definitions of “success” varied. In line with SDM theory, almost all recruiters focused on a patient-centered definition, citing that the interaction provides patients with more knowledge of their illness and available studies (*n* = 5) and builds rapport between recruiter and patient, which may improve recruitment for future studies (*n* = 3). Three recruiters also talked about personal success; two identified that despite patient success, the interaction might be personally unsuccessful regarding enrollment (*n* = 2), while another identified personal success with non-enrollment in terms of learning which patients are more likely to participate.

An essential component to SDM is taking a non-directive approach to the decision-making process, providing patients with balanced information on all options available, as opposed to a directive approach where the chief goal is to enroll patients. Most recruiters reported they took a non-directive or mixed approach, indicating they try to provide patients with the information needed to make an informed decision and guide them in making a decision they are comfortable with. Three indicated they take a more directive approach in promoting participation (but stressed being non-coercive). One recruiter indicated their directive approach was specific to the SAVER trial, because they viewed the study as minimal risk. For higher-risk studies, they reported a more non-directive approach to help patients weigh the risks and benefits of participating.

Core constructs in a SDM approach include verifying patient understanding, providing information about all options, assessing patient’s decision-making needs, clarifying patient attitudes and values, providing structured guidance, clarifying patient’s outcome preferences, discussing their self-efficacy, and building their skills in deliberating, communicating, and assessing support [20]. We asked recruiters how frequently they felt they addressed these constructs in an informed consent interaction. Responses were coded into three categories (never/rarely, sometimes, usually/always; Table 2). The components of SDM recruiters reported they regularly used included assessing patient’s decision-making needs, ensuring they understood what they were consenting to, providing information on their options (both harms and benefits to participating or not), facilitating the decision-making process, and defining the decision (i.e. trial participation). This was typically done through a two-way conversation where recruiters encouraged patients to ask questions, and recruiters likewise asked questions to ensure understanding.

**Table 2:** Study recruiter responses to queries about the use of elements of shared decision making during interviews (*n* = 9). Responses organized by element, response, and frequency

SDM question	***N***	Representative quote
Can an informed consent interaction be successful if it does not result in enrollment?	9 - Yes	“Oh yes, absolutely. There has been many times where we have gone through lots of information and the patient does not end up going into the study, but during that time I’ve been able to answer a lot of questions about possibly the disease or about the drug.”
		“…yeah the patient probably leaves with a little bit more information about their diagnosis. They, they often ask me maybe questions that they forgot to ask the doctor and then I can bring those up with the doctor before they go home…So yeah as far as the patient yeah there is some, there is success for them, not for me for enrolling…”
Would you describe your style in informed consent interactions as more directive or non-directive?	5 - Non-directive	“I would say non-directive I would say more help them with the decision on whether or not they, you know, will participate. So it’s just to inform them as much as possible about, about the study…”
	3 - Directive	“I’m probably more directive. I spend a lot of time highlighting potential benefits to the patient. Never, you know, giving false promises or anything like that but as we mentioned with me having drank the Kool-Aid I have that approach when I talk to the patient… So I take that more kind of that approach versus just being totally impartial. There is some definite biases into the project but never in a coercive way.”
	1 - Both	“I’d say it’s a combination to be honest with you… I would say at the beginning it’s kind of directive when I first start talking to the patient. You know maybe my wording will be more directive… so you more use like salesman words… but then I do not want to pressure the patient so I’m, I’m pretty, indirect.”
**How often would your interactions with patients involve these activities:**		
Assessing the decision making needs of the patient?	8 - Always/usually 1 - Sometimes 0 - Rarely/never	“I would say pretty much every time; because I mean I’m asking them if they have questions and encouraging it and, you know, asking them to speak either with their family doctor or their family or to do their own Googling themselves to see, you know, what other literature exists out there.”
Verifying understanding?	6 - Always/usually 3 - Sometimes 0 - Rarely/never	“Yeah like we ask them questions like if they for example if they know the signs and symptoms of clots and ask them to relay that back to us...”
Providing information on their options, benefits, and harms (e.g., verbally or with additional patient education resources)?	9 - Always/usually 0 - Sometimes 0 - Rarely/never	“So every time in addition to it being incorporated into the pitch that I have for the study it’s also all laid out in the consent form. There’s the section for side effects, risks and benefits… what would happen if they did not participate. So they are getting, you know, something written down but then it’s also mentioned to them as part of the pitch.”
Clarifying their values and their attitude/tolerance towards risks?	4 - Always/usually 3 - Sometimes 1 - Rarely/never 1 – No response	“…I think I do that every time like because I do talk about the risks and ... Here’s the risk of bleeding if you stay on. Here’s the percentage of people that get blood clots. Here’s the percentage, right, so which risk factor are you more happy to, you know, can you live with the risk factor of a blood clot versus can you or would you rather stay on the drug and have this risk factor of a major bleed?”
		“So if I’m understanding it correctly, I would say sometimes. So if they focus on a specific side effect maybe that’s listed. I try to quantify it for them a little bit because we have to list every possible side effect that’s ever been experienced, you know, like let them know that that’s the case that it does not mean that that’s actually gonna happen to you. And if it does happen to you here’s the plan we have in place and how we’ll deal with it. And, you know, maybe give them kind of information on the percentage of patients that could actually experience that side effect.”
Building their skills in deliberating, communicating, and assessing support?	1 - Always/usually 1 - Sometimes 7 - Rarely/never	“It’s not our job to build those kinds of skills with patients…”
Facilitating progress towards decision-making?	7 - Always/usually 1 - Sometimes 1 - Rarely/never	“Oh I’d say usually then I guess … it does not mean I’m like pushing them to say a certain answer it just means I’m helping them make their answer.”
Discussing their ability/self-efficacy?	5 - Always/usually 1 - Sometimes 3 - Rarely/never	“Mm hmm well yeah and we do. And one of the things that we specifically talk about when we talk about coming into the research study is that this is 100% voluntary. That, you know, by signing consent you are not locked into anything, right.”
		“No I, I do not discuss that stuff specifically because I worry about it maybe in potentially being like condescending or something.”
Defining/explaining the decision?	8 - Always/usually 0 - Sometimes 0 - Rarely/never 1 - no response	“… we go through all the information and sometimes they’ll say, can I think about it overnight or, you know, I want to talk to my wife about it. And what I would say to them, you know, unfortunately for this type of study we have to do it today. So if you are not comfortable and I do tell people if you are coming in a research study I want you 100% into the study. I want you to, to be coming into the study, you know, that you are fully confident coming in, right. If you are thinking if you have any doubts do not go in the study and I tell them that.”

Other components of SDM were used less frequently by recruiters; clarifying the values of patients and their attitudes towards risk and discussing the patient’s abilities/self-efficacy in making a decision. Some recruiters indicated that when patients showed concern over side effects or other issues, they addressed these concerns with further education and clarification. If recruiters were unsure of a patient’s capacity to make an informed decision on their own, they would recommend involvement from family members to help in the decision-making process. Finally, the majority of recruiters indicated they do not specifically work to build the skillset of patients in deliberating, communicating, or assessing support. This is generally not seen as part of their job; however, some recruiters indicated they address this component by validating patients’ concerns, reassuring them that participation is voluntary and working with them to identify supportive people to help with their decision.

## Discussion

Previous efforts to improve trial recruitment have rarely used theory-based approaches. Even large-scale initiatives such as the studies within a trial repository [41] largely include studies with interventions focused on a single aspect of recruitment that lack a theoretical basis. This study is the first to bring the complementary theoretical lenses of SDM and TDF to interviews seeking to understand how to improve clinical trial recruitment.

Overall, recruiters reported practices consistent with many elements of SDM, despite having no formal training in this area. These recruiters see their recruitment interactions as potentially successful even if it does not result in enrollment. While the definition of success tends to vary from person to person, they generally focus on aspects of patient experience, true to SDM theory. There is considerable variation in the amount of “directiveness” different recruiters endorse using in the interaction. Further work should explore whether observational techniques also show this variation and whether these differences vary with recruiting effectiveness and patient experience. It may also be important to examine the degree to which directiveness varies across different kinds of trials and recruiters.

Core SDM elements were frequently followed by these recruiters. Almost all report assessing the decision-making needs of the individual as important to their role. This important aspect of recruiting is rarely addressed by informed consent documentation or assessed by ethics boards. Many also reported verifying patient understanding, but there is considerable variation in how recruiters do this, from relatively passive (looking at body language) to more active efforts (testing knowledge). There is no guidance in the literature as to which of these techniques should be encouraged. It appears that, in general, recruiters are trying to implement many aspects of SDM, elements that are not well addressed by informed consent documents [20, 21]. However, they are doing it without any formal training, leading to great variability in how often and how well these constructs are addressed.

Ethical implications of applying the principles of SDM to clinical trial participation decisions require more study. Research ethics boards require that participants be fully informed and understand the risks and benefits of participating in a clinical trial. SDM can take this requirement one step further, by placing recruiters in a key role of working with participants to arrive at a participation decision that is both informed and consistent with their values. Our study has demonstrated that these interactions are already taking place during informed consent interactions informally and to a varying degree. This SDM perspective suggests that the informed consent process may be enhanced by formally training recruiters in using the principles of SDM, thus leading to individuals making participation decisions better in line with their own values and health outcome preferences. This in turn could lead to higher patient satisfaction and lower dropout rates in clinical trials. While there has been considerable work done on SDM training for healthcare providers, none have been specifically designed for trial recruiters [42]. Future work might develop training materials for SDM in trial recruitment and evaluate their effectiveness in improving the informed decision-making process for patients.

We were able to ask questions relevant to each TDF domain in a way that recruiters understood, showing the feasibility of applying this framework in this new context. These questions provided a framework to guide discussions about their recruitment barriers and drivers, yielding a more comprehensive set of factors to consider than previous bottom-up efforts. For example, Kurt et al. [43] focused heavily on social influences, Jones et al. [44] primarily examined consequences, and Frew et al. [45, 46] took a socioecological approach, again focusing on social influences and roles. While these findings have provided insight into some of the barriers and drivers to trial participation, they do not consider the full range of domains that can influence behavior. Our work suggests that the TDF may provide a feasible and theory-informed way to categorize barriers and drivers to trial participation and to design recruitment strategies that more effectively address them (see also Sheridan 2020) [47].

Our aim to identify the full range of barriers and drivers relevant to study recruitment in the target trial according to the TDF was supported by our findings. Results showed that nearly all TDF domains were seen as relevant to trial recruitment behaviors, further supporting the notion that recruitment is a more complex problem than typically considered. The Behavioral Regulation and Social Influences domains have been widely studied, and recruiters clearly thought they were important. Other domains however (e.g. Skills, Nature of the Behavior) are not evidently considered in many of the existing recruitment survey instruments [43–45], yet elicited important barriers and drivers in our discussions. Other domains were mentioned relatively infrequently (e.g. Social/Professional Role & Identity, Emotion); whether these domains are generally not a factor in recruitment situations, or become more important in other recruitment situations, is a question for further work.

A key benefit in applying a TDF approach is that it suggests specific ways to overcome the barriers identified. Although a wide range of factors were identified from the current study, categorizing them based on domains allows multiple factors to be targeted by specific behavior change interventions. Michie et al. [26] developed and recently updated [40, 48] a matrix linking TDF domains to behavior change techniques based on consensus and previous literature [40, 48]. From this matrix, the most promising behavior change interventions for the SAVER trial include problem-solving (Behavioral Regulation; Beliefs about Capabilities), social support (Social Influences; Environmental Context/Resources), instruction on how to perform the behavior (Knowledge; Skills; Beliefs about Capabilities), goal setting and feedback (Motivation and Goals), and restructuring the environment (Environmental Context/Resources). Based on this matrix, we were able to develop a report for the principal investigators of the SAVER trial providing specific recommendations for improving recruitment in the main trial. Considering the novelty of our approach, any implemented changes should be evaluated for feasibility and effectiveness. Such approaches could be used to develop more formal training for recruiters for future phases of the SAVER trial at additional sites or indeed for other clinical trials.

### Limitations and future directions

Study limitations include issues related to sampling, saturation, and participant understanding. Our results and conclusions are based on information from individuals recruiting for a single trial employing non-physician recruiters. Similar interviews conducted with physician recruiters may reveal other barriers and drivers, as some of our key findings related to coordination between team members, including physician buy-in. Similarly, interviewing recruiters for other trials may reveal other important barriers and drivers. Research involving more diverse samples would provide further insight into which TDF domains and interventions are relevant to a broad range of studies and recruiters.

Our study was limited to a small sample frame of recruiters from a single multisite study. While we interviewed all active recruiters for the study, typical TDF studies follow the 10 plus 3 rule for data saturation (i.e. conduct 13 interviews, if no new themes in the last three, stop) [39]. An examination of the data reveals only one new sub-theme emerged from the final three interviews, suggesting that while it is possible that additional interviews would have revealed new themes, most were likely identified for this group. Findings from this initial study using a single trial may set the stage for future studies that could apply the TDF and SDM frameworks to a wider range of professionals across diverse research setting to examine the generalizability of our findings.

Individual themes, barriers, and drivers identified with these interviews may or may not be directly associate with recruitment effectiveness; they constitute self-reports of what recruiters believed to be effective strategies. Future research should evaluate whether the identified factors affect recruitment and patient satisfaction in active trials.

Finally, the SDM literature uses language and concepts that may be unfamiliar to untrained individuals. While most recruiters endorsed using many SDM concepts, there was apparent variation in their level of understanding. Recruiters sometimes asked for clarification, indicating a lack of familiarity with some concepts. For example, one recruiter indicated they use a non-directive approach, but proceeded to describe a fairly directive process (i.e. one-sided, focusing on the benefits of participating). Future research should evaluate actual SDM knowledge as part of the interview process and further refine interview language to clarify these concepts for the population of interest.

## Conclusions

The results demonstrate the utility of applying the TDF and SDM lenses to shed new light on the challenges of recruiting for clinical trials. These approaches revealed new concepts to apply to this issue. The core concepts in SDM are already being used in an ad hoc manner with varying degrees by study recruiters, which could be improved by providing standardized training and guidance in this area. Using a TDF approach to barriers and drivers revealed a variety of factors not identified by previous efforts. The TDF framework also provides clear approaches to addressing these factors when designing recruitment interventions. Future research should evaluate these interventions in their ability to improve study recruitment and patient decision-making for clinical trials.

## Supplementary Information

**Additional file 1.** Contains the COREQ checklist, including page references.

**Additional file 2.** Contains the study interview guide used during interviews with recruiters.

## Data Availability

The data generated and used during the current study are available from the corresponding author on reasonable request.

## Abbreviations

SDM: Shared decision-making
TDF: Theoretical Domains Framework
SAVER: StAtins for Venous Event Reduction study
