# Supplementary material for: Using behavioral theory and shared decision-making to understand clinical trial recruitment: interviews with trial recruiters
Source: Trials. 2021 Apr 21;22:298. doi: 10.1186/s13063-021-05257-x (PMC8058968; doi:10.1186/s13063-021-05257-x)
Supplement: Supplementary file 1 — Additional file 1. Contains the COREQ checklist, including page references. [file 13063_2021_5257_MOESM1_ESM.docx]

**Additional File 1**

**Table 1.** Consolidated criteria for reporting qualitative studies (COREQ): 32-item checklist [[1](#_ENREF_1)]

| No | Item | Description | Page reference |
| --- | --- | --- | --- |
| Domain 1: Research team and reflexivity | | | |
| 1 | Interviewer/ facilitator | All interviews were conducted by KC | Page 7 |
| 2 | Credentials | The interviewer was a Master’s level researcher | Page 7 |
| 3 | Occupation | The interviewer was a research coordinator at the Ottawa Hospital Research Institute | Page 7 |
| 4 | Gender | The Interviewer was female | Page 7 |
| 5 | Experience and training | The interviewer had over 10 years of experience in conducting interviews/qualitative research | Page 7 |
| 6 | Relationship established | The interviewees were active trial recruiters for MR. No relationship existed between the interviewer and interviewees prior to the study | Page 6 |
| 7 | Participant knowledge of the interviewer | Participants were provided with an initial notification (from MR) and formal invitation (from JCB) to the interview, including a participant information document describing the study | Pages 6-7 |
| 8 | Interviewer characteristics | The interviewer (KC) had a pre-existing interest in recruitment interventions, as well as shared decision making and the TDF. The larger study team have published extensively on these theoretical frameworks | n/a |
| Domain 2: Study design | | | |
| 9 | Methodological orientation and Theory | The theoretical frameworks of shared decision-making and the Theoretical Domains Framework were used | Page 4 |
| 10 | Sampling | A purposive sample of study recruiters was selected | Page 6 |
| 11 | Method of approach | Participants were approached via e-mail | Page 6 |
| 12 | Sample size | The final sample included 9 out of 10 possible study recruiters | Page 8 |
| 13 | Non-participation | One participant was not eligible due to a change in employment | Page 8 |
| 14 | Setting of data collection | All interviews were conducted either over the phone or in person in a private room at an academic hospital | Page 7 |
| 15 | Presence of non-participants | Only the interviewer and participant were present in each interview (i.e. one-on-one) | Page 7 |
| 16 | Description of sample | The sample included 9 participants (67% female) with 3 to 30 years of experience in trial recruiting | Page 8 |
| 17 | Interview guide | The interview guide is attached (Additional file 2). It was pilot tested among 3 non-participant recruiters | Page 6;  Additional file 2 |
| 18 | Repeat interviews | No repeat interviews were conducted | n/a |
| 19 | Audio/visual recording | Interviews were digitally, audio recorded | Page 7 |
| 20 | Field notes | Field notes were made during the interviews | Page 7 |
| 21 | Duration | Interviews ranged from 42 to 79 minutes in length (m = 63) | Page 8 |
| 22 | Data saturation | Data saturation was nearly reached with only one new theme arising in the final 3 interviews | Page 15 |
| 23 | Transcripts returned | Transcripts were not verified by participants | n/a |
| Domain 3: Analysis and findings | | | |
| 24 | Number of data coders | Two coders (CLV and KC) coded all interviews | Page 7 |
| 25 | Description of the coding tree | Sub-themes were grouped according to categories of action | Page 8;  Table 1 |
| 26 | Derivation of themes | For SDM coding, themes were identified in advance. For TDF coding, themes were developed inductively with the first two interviews and modified throughout coding of the other seven interviews | Page 7 |
| 27 | Software | Data extraction and coding was completed using Microsoft Excel | Page 7 |
| 28 | Participant checking | Participants did not provide feedback on the findings | n/a |
| 29 | Quotations presented | Quotes are presented to illustrate each theme. Participant numbers were not used to avoid the risk of re-identification in a small sample | Table 1;  Table 2; |
| 30 | Data and findings consistent | Results presented are linked directly with the findings/interpretation of data | Pages 8-15 |
| 31 | Clarity of major themes | The major themes and sub-themes are discussed in detail in the results section | Pages 8-12 |
| 32 | Clarity of minor themes | Sub-themes and TDF/SDM domains not covered in our themes are discussed in the results and discussion sections | Pages 8-15 |

References:

[1]. Tong A, Sainsbury P, Craig J. Consolidated criteria for reporting qualitative research (COREQ): a 32-item checklist for interviews and focus groups. .Int J Qual Health Care 2007;19(6):349-57.
