# Supplementary material for: Using behavioral theory and shared decision-making to understand clinical trial recruitment: interviews with trial recruiters
Source: Trials. 2021 Apr 21;22:298. doi: 10.1186/s13063-021-05257-x (PMC8058968; doi:10.1186/s13063-021-05257-x)
Supplement: Supplementary file 2 — Additional file 2. Contains the study interview guide used during interviews with recruiters. [file 13063_2021_5257_MOESM2_ESM.docx]

**Appendix - Interview Guide**

**Improving Participant Recruitment and Retention in Thrombosis Research: Understanding what Study Recruiters do.**

Thanks for taking the time to meet with me. We are interested in better understanding the recruitment process for studies like the SAVER trial, and are interested in your experience in recruiting patients into trials like this. The scientific literature on study recruitment has focused primarily on how to boost recruitment rates, rather than how the recruitment process contributes to patient experience with trial participation. It has also rarely explored the expertise of people who do a lot of recruiting for large-scale trials. So the plan for this interview is to explore the things that you think are important for successful recruitment interviews, what are the things to avoid, and what you strive to achieve when you are in the room with a particular patient.

Our scientific team is interested in general lessons about recruiting to trials, so feel completely free to be open and honest about your views. No released information will ever be able to identify you or anyone you talk about. Reports stemming from this work will only use unidentifiable quotes, or describe the discussions in aggregate form. I have a series of questions here, and I may ask for some points of clarification throughout just to make sure I understand your process, but feel free to mention whatever you feel is relevant at any time. We are interested in how you go about these interactions, any problems you run into, how you solve them, and any recommendations you might have to others doing the same thing.

Any questions before we start? OK if I record our conversation so that I can review it later?

PART 1: Shared Decision Making

1. How long have you been consenting for clinical trials of one sort or another?
2. What are some examples of trials that you have consented patients for?
3. How much of your effort is spent identifying eligible patients, as opposed to consenting those already identified as eligible?
4. Can you talk me through your usual approach to consenting a patient in a clinical trial?” [*Probe: “How do you approach them?”, “Are they referred to you?”]*
5. In your view, can an informed consent interaction be successful if it didn’t result in enrolment? Can an interaction be a failure if it DID result in enrolment? *[Prompt: why or why not?]*
6. What do you think the most important factors are in determining whether a person will consent to participate in your study? *[Prompt: Factors related to the recruiter, the study, the patient?]*
7. What do you think are the most important factors in determining whether a patient clearly understands the study they are being asked to participate in?
8. What do you think are the most important factors in whether a patient will be satisfied with the overall informed consent process? *[Prompt; satisfied with the interaction, their interactions with the study team, the documents…]*
9. Can you think of any specific problems you have run into when conducting informed consent interactions? *[Prompt: How did you solve them?]*

PART 2: Theoretical Domains of Behavior Change

For the rest of our discussion, I’d like you to think specifically about recruiting patients into the SAVER trial. Some of these questions may seem a little repetitive, but please bear with me, as I want to be thorough and make sure that I fully capture your views. Is that ok?

**Nature of the behaviour:**

How much of your position involves recruiting patients to research studies? To the SAVER study?

What materials do you typically use when consenting a patient to the SAVER study?

**Knowledge:**

What information do you think is essential for patients to know in order to successfully consent them into the study?

**Skills:**

Have you ever received any formal training in consenting patients into the SAVER study? Into trials LIKE the SAVER study (if yes: what did that consist of?; if no: Do you think you might benefit from any if it were offered?)

Have you had any more general formal training in interviewing, talking with patients, etc.?

Do the patients need to have any specific skills before you’ll be able to successfully consent them?

How is the recruitment conversation affected if they don’t have these skills?

**Social/Professional Role and Identity:**

To what extent do you see consenting for trials as part of your current professional role?

Are there other colleagues that play important roles in consenting your patients?

*Prompt: Who? What are those roles?*

**Beliefs about Capabilities:**

How confident do you feel that you can consent enough of your patients into the SAVER trial?

Are there any situations or factors that make you feel less confident in your ability to consent patients? What might improve your confidence?

**Beliefs about Consequences:**

- What are some of the benefits of you consenting patients to the SAVER trial? *(Prompt: benefits for self, to others, to the trial)*
- Are there any negatives of you successfully consenting patients to the trial? *(Prompt: consequences to self, to others, to the trial)*
- What would happen if you were unable to successfully consent patients to the trial? *(Prompt: consequences to self, to others, to the trial)*
- Do these issues ever come to mind or influence how you approach the consenting process? If so, which ones?

**Motivation and Goals**

- How motivated are you to recruit patients to the SAVER trial? How much of a priority is it compared to everything else you do? What are the higher priorities and how do you fit this in?
- What are you trying to achieve as the goals of the consent interviews? What are you trying to avoid, if anything, with the consent interviews? *(Prompt: goals for self; goals for the trial)*
- Are their specific targets or quotas that you need to meet?

**Memory, Attention, and Decision Processes**

- Might you ever decide NOT to try to recruit someone who was eligible for the SAVER trial? Why?
- Are there any circumstances where you might FORGET to recruit someone? When? Are there any situations were reminders might be of benefit?

**Environmental Context and Resources**

- Do you have the resources required to consent for the SAVER trial?
- What aspects of your work environment HELP you with consenting?
- What aspects of your work environment HINDER you with consenting?

**Social Influences**

- Who expects you to recruit for the SAVER trial? *Prompt: anyone else?*
- Of those people, who influences you the most?
- Do you have any other colleagues that are influencing/aspiring you?

**Emotion**

What emotions come to mind when you think about recruiting a patient to this trial? *Prompt: Are there emotional components to this recruitment process?*

**Behavioural Regulation**

Do you receive any feedback about your consenting? What form does it take?

Can you think of any new procedures or strategies that might help you consent more effectively? *Prompt: maybe some that have worked better elsewhere that you might want to implement here?*

1. Would you describe your style in informed consent interviews as more directive (want to get them to participate) or non-directive (want to help them with the decision)?
2. Finally, the next questions derive from elements from the shared decision making literature. How often would you say that your interactions with your patients involve the following activities? [You may have already touched on some of these already]. So, we’d like you to tell us which of the following activities do you do and which seem foreign.

|  | **Never** | **Rarely** | **Sometimes** | **Usually** | **Always** |
| --- | --- | --- | --- | --- | --- |
| 1. Assessing the decision making needs of the patient?   - what they need to make the decision – all the information/knowledge, other people, etc. | □ | □ | □ | □ | □ |
| 1. Verifying understanding?   - quizzing them on the information? | □ | □ | □ | □ | □ |
| 1. Providing information on their options, benefits, and harms (e.g., verbally or with additional patient education resources)?  - Making it clear what their options/choices are. | □ | □ | □ | □ | □ |
| 1. Clarifying their values, and their attitude/tolerance towards risks?   - Clarifying the value or importance of the benefits and risks of participating or not participating. | □ | □ | □ | □ | □ |
| 1. Building their skills in deliberating, communicating, and assessing support?   - Taking them thru the steps/breaking down the decision making process. | □ | □ | □ | □ | □ |
| 1. Facilitating progress towards decision making? | □ | □ | □ | □ | □ |
| 1. Discussing their ability/self-efficacy?   - discussing if they feel they have control over their decision? | □ | □ | □ | □ | □ |
| 1. Defining/explaining the decision? | □ | □ | □ | □ | □ |

Is there anything else that comes to mind in terms of your experiences with recruiting patients to clinical studies?

**Thank You!**
